# A novel diffuse liver nodule detector via integrating semantic edge features and probabilistic uncertainty modeling

**DOI:** 10.3389/frai.2026.1801342

**Published:** 2026-04-20

**Authors:** Lei Tian, Xiang Liu, Yunyu Shi, Yu Ji, Shuohong Wang

**Affiliations:** 1 School of Electronic and Electrical Engineering, Shanghai University of Engineering Science, Shanghai, China; 2 Department of Cell Biology and Harvard Medical School, Boston, MA, United States

**Keywords:** Dirichlet evidential theory, liver nodule, medical ultrasound, probability-guided attention, semantic edge

## Abstract

**Introduction:**

Ultrasound image segmentation of diffuse liver fibrosis nodules confronts three critical challenges: boundary ambiguity caused by gradual tissue transitions, texture heterogeneity arising from fibrotic variations, and inadequate uncertainty quantification, which manifests as overconfident misclassifications at fibrotic nodules.

**Methods:**

To address these challenges, this paper proposes the Edge-Semantics Probabilistic Dirichlet Network (ESPD-Net), which integrates Dirichlet evidential theory into diffuse lesion segmentation for nodule detection. ESPD-Net employs three synergistic components: (1) The Semantic-Probabilistic Dual Path Fusion (SPDF) bottleneck constructs parallel semantic and probabilistic pathways to capture local morphological and global distribution features. (2) The Dirichlet Evidential Guided Decoder (DEGD) reformulates segmentation as second-order probabilistic modeling under evidential theory, guiding the adaptive feature decoding process by outputting calibrated uncertainty distributions. (3) Guided by these localized high-uncertainty regions, the Dirichlet Boundary Aware Refinement (DBAR) module executes targeted geometric corrections to precisely align ambiguous lesion boundaries.

**Results:**

Evaluations on murine and clinical datasets demonstrate that ESPD-Net significantly outperforms state-of-the-art methods. Specifically, it achieves a Dice of 0.855 (+0.043) and an IoU of 0.747 (+0.057). Furthermore, the model effectively minimizes calibration and boundary errors, reducing ECE to 3.85% and HD95 to 3.25.

**Discussion:**

These findings demonstrate that the proposed ESPD-Net effectively addresses the challenges of diffuse lesion segmentation, thereby objectively confirming its clinical potential for computer-aided diagnosis.

## 1 Introduction

Liver fibrosis is a critical period in the progression of chronic liver disease, characterized by excessive accumulation of extracellular matrix proteins after persistent hepatocyte injury (Zhang et al., 2025). Accurate assessment of fibrosis and early identification of potential dysplastic nodules are the key to preventing the occurrence of hepatocellular carcinoma and guiding clinical intervention (Kisseleva and Brenner, 2021). Despite advancements in diagnostic modalities and computational algorithms yielding notable progress, achieving accurate segmentation of diffuse liver fibrosis nodules remains a significant challenge (Zhou et al., 2020).

Unlike benign tumors with distinct capsules, diffuse fibrotic nodules exhibit highly complex pathological morphological features on ultrasound images (Shuo et al., 2016). First, structural remodeling of the hepatic parenchyma leads to the formation of extensive regenerative nodules (RNs). These nodules, arising from the compensatory proliferation of hepatocytes to replace lost parenchyma, are surrounded by collagenous septa, macroscopically presenting a heterogeneous “pebble-like" appearance (Lyshchik et al., 2024). The vast majority of such nodules are benign, however, some regenerative nodules accumulate genetic mutations through chronic injury-repair cycles, ultimately transforming into dysplastic nodules (DNs) or even early hepatocellular carcinoma (HCC) (Tan et al., 2021). Since DNs are frequently embedded within morphologically indistinguishable RNs and an irregular fibrotic background (Choi et al., 2021),the primary clinical challenge is to precisely localize these potentially malignant targets amidst severe acoustic clutter.

At the computational level, this target extraction is further severely hindered by the lesions' geometric and acoustic complexities. Thus Traditional algorithms often exhibit oversegmentation or undersegmentation when processing such data (Senthilkumaran and Vaithegi, 2016; Zhou et al., 2016; Chen and Pan, 2018; Liu et al., 2017). Geometrically, the lack of a clear acoustic interface results in gradual gradient transitions, rendering conventional edge detection operators and shape-prior models largely ineffective (Wu S. et al., 2024). Acoustically, significant textural heterogeneity and grayscale overlap with background tissues disrupt traditional region based algorithms (Wang et al., 2016; Zhou et al., 2025; Mishra et al., 2018). While U-Net and its variants have demonstrated remarkable success in medical image analysis, achieving accurate segmentation for lesions with well-defined boundaries, they are highly susceptible to producing overconfident and overfitted predictions at the ambiguous interfaces of diffuse nodules (Liu et al., 2020). The fundamental limitation is that traditional networks output categorical probabilities as deterministic point estimates, failing to quantify the underlying reliability of these predictions,making the network to overfit to local acoustic noise and compromising its generalization ability. Therefore, effectively quantifying predictive uncertainty and utilizing it to guide geometric boundary refinement provides a new pathway for the accurate segmentation of diffuse lesions (Lyshchik et al., 2024; Huang et al., 2024).

To address the aforementioned challenges, this paper proposes ESPD-Net, a diffuse nodule detector that integrates nodule edge semantics with uncertainty probability feature modeling, aiming to achieve precise segmentation of liver nodules in complex backgrounds. The main innovations are summarized as follows:

- **Semantic-probabilistic dual-path fusion (SPDF) bottleneck**: the SPDF module employs deformable convolutions to capture irregular morphological features, while simultaneously utilizing a Transformer to construct a semantic attention mechanism guided by probability density. By dynamically recalibrating feature weights, this design effectively mitigates the interference caused by significant grayscale overlap, reducing the dependency of conventional methods on strict feature separability. The dual-path extracted features supply the requisite semantic context for the DEGD module and the feature priors essential for the DBAR module.
- **Dirichlet evidential-guided decoder (DEGD)**: the DEGD module systematically incorporates Dirichlet evidential theory into the task of liver nodule detection. It reformulates the segmentation decoding stage as an evidence accumulation process.By generating Dirichlet concentration parameters via a multi-scale evidence pyramid, the module achieves second-order uncertainty quantification,which utilize these calibrated uncertainty distributions to guide feature decoding. This mechanism ensures that the network adaptively filters unreliable signal propagation, effectively distilling precise and deterministic lesion representations from highly ambiguous fibrotic backgrounds.
- **Dirichlet boundary-aware refinement (DBAR)**: the DBAR module synergistically integrates traditional gradient cues with deep semantic edges. Leveraging the uncertainty information generated by the DEGD as a prior, the module adaptively directs attention toward ambiguous yet critical tissue interfaces, progressively correcting prediction results through an iterative refinement mechanism.

## 2 Related work

### 2.1 Development of deep learning segmentation architecture

The emergence of deep convolutional neural networks has fundamentally transformed the paradigm of medical image segmentation. The advent of fully convolutional networks (Long et al., 2015) firstly enabled end-to-end pixel-level classification. Building upon this, the U-Net architecture characterized by its symmetric encoder-decoder structure and skip connections (Ronneberger et al., 2015), established the foundational paradigm for this field. However, constrained by the local receptive field inherent in purely convolutional operations, these early approaches frequently suffer from fine-grained feature loss (Liu et al., 2019). When processing minute lesions or complex structures, they struggle to delineate ambiguous gray level differences between pathological and normal tissues (Ma et al., 2016; Zheng et al., 2023).

To overcome these local limitations, the research focus shifted toward architectures capable of capturing broader contextual information. Approaches like DSRU-Net (Zhou et al., 2019) incorporated dense and residual connections to enlarge receptive fields. Subsequently, inspired by the success of Vision Transformers, models like TransUNet (Chen et al., 2021) integrated self-attention mechanisms to explicitly model global dependencies. To further mitigate the prohibitive computational complexity of pure Transformers on high-resolution medical images, efficient alternatives such as the MLP-based UNeXt (Valanarasu and Patel, 2022) were introduced. Building on these advancements, hybrid dual-path architectures have emerged as a dominant trend. Models such as Dense-PSP-UNet (Ansari et al., 2023) and dual-path RNN frameworks (Song et al., 2023) successfully synergize the localized precise feature extraction of CNNs with the global awareness of advanced attention mechanisms.

While these architectural innovations enhanced feature representation capabilities, limitations remain regarding boundary localization for diffuse lesions and the quantification of prediction reliability. Most existing fusion and skip-connection strategies rely on simple concatenation or uncalibrated weighted summation.They fail to utilize uncertainty information to dynamically adjust feature propagation weights, resulting in the indiscriminate propagation of unreliable features and leading to error accumulation in regions with ambiguous boundaries.

### 2.2 Attention mechanism and boundary semantics

Beyond architectural backbones, the integration of attention mechanisms has significantly advanced the capability of segmentation networks to selectively model critical features. Roy et al. (2018) proposed the concurrent Spatial and Channel Squeeze module to simultaneously model channel-wise and spatial dependencies. Oktay et al. (2018) introduced Attention U-Net, utilizing attention gate mechanisms to filter encoder features and suppress interference from irrelevant regions; this selective propagation demonstrated significant superiority in organ segmentation.

Addressing the boundary ambiguity inherent in ultrasound images. Mishra et al. (2018) proposed a deeply supervised boundary attention network specifically designed to explicitly model edge regions. Wang et al. (2022) developed a Boundary-Aware Context Neural Network that jointly optimizes segmentation and boundary detection via multi-task learning. Furthermore, Wu J. et al. (2024) designed BACA-Net, employing a boundary attention module to enhance edge features and improve the localization of liver contours. Most recently, Qin et al. (2025) proposed MBE-UNet, which refines boundary quality through a multi-branch enhancement strategy.

Despite their progress in boundary localization, these methods operate exclusively within deterministic prediction frameworks. By treating boundary detection as a fixed classification or regression problem, they fundamentally fail to distinguish between genuine structural edges and regions of high epistemic uncertainty. In diffuse liver fibrosis nodules, gradual tissue transitions render boundaries intrinsically ambiguous. Consequently, the overconfidence typical of these deterministic boundary enhancement approaches forces the model to fit localized acoustic noise, potentially leading to clinical misdiagnosis (Zhou and Chen, 2024).

### 2.3 Uncertainty quantification approaches

In traditional medical image segmentation, although Bayesian neural networks, Monte Carlo Dropout algorithms (Akram et al., 2025), and deep ensembles typically perform well by estimating uncertainty through sampling deterministic outputs,they introduce excessive computational overhead and damage feature information due to their inherent dependence on multiple forward passing or independent model training.

To address these computational bottlenecks, Evidential Deep Learning (EDL) (He et al., 2025) has been introduced to model second-order uncertainty in a single forward pass. By placing a Dirichlet distribution over the class probabilities, EDL formulates classification as an evidence accumulation process. The total uncertainty is formally defined as $U=K/\overset{K}{\underset{k=1}{\sum }}\alpha_{k}$, where **α** denotes the evidence strength. Nevertheless, existing evidential segmentation baselines primarily utilize EDL as an auxiliary loss function for post-prediction calibration. In these frameworks, the uncertainty estimation process remains functionally decoupled from the network's internal feature representation.

Unlike prior studies that treat uncertainty solely as a terminal output or a loss penalty,the ESPD-Net internalizes evidential theory directly into the architectural design. Rather than relying on *post hoc* calibration,it translates theoretical uncertainty distributions into intermediate feature guidance and achieves detection segmentation by embedding uncertainty modulation into the decoding and refinement stages.

## 3 Materials and methods

This paper proposes an uncertainty aware framework based on Dirichlet evidential theory for the ultrasound segmentation of liver fibrosis nodules. By integrating edge semantic features with probability density modeling, this method improves localization accuracy at highly blurred lesion boundaries and effectively adapts to the diffuse distribution characteristics of fibrotic lesions, thereby providing valuable segmentation results to assist in clinical diagnosis.

### 3.1 Datasets and ethical approvals

To evaluate the proposed ESPD-Net, we utilized two completely independent ultrasound datasets: a clinical liver fibrosis dataset and a mouse liver fibrosis dataset. Each dataset was individually partitioned into corresponding training, validation, and testing sets at the level of individual patients or mice, following a strict 7:1:2 ratio. The mouse dataset, which covers the complete pathological cycle of liver fibrosis, serves as the primary benchmark for fundamental feature learning and internal validation. The clinical dataset provides a more challenging benchmark for evaluating the clinical robustness and stability of the architecture.

- **Historical mouse liver fibrosis ultrasound dataset:** this dataset comprises 344 ultrasound image sets and corresponding nodule masks from drug-induced liver fibrosis mouse models, provided by Shanghai Changzheng Hospital. Spanning the complete pathological cycle from early-stage fibrosis to late-stage cirrhosis, these images constitute historical archival data. This study is strictly a retrospective algorithmic analysis of existing images; no new *in vivo* animal experiments were conducted, and the original data acquisition adhered to standard animal care guidelines.
- **Clinical ultrasound validation dataset:** this dataset consists of 67 clinical ultrasound samples from Shanghai Changzheng Hospital, supported by explicit clinical pathological results. This retrospective study complies strictly with the Declaration of Helsinki and was formally approved by the Biomedical Research Ethics Committee of Shanghai Changzheng Hospital (Approval No. 2017SL013). Because the study utilized strictly de-identified and anonymized images, the ethics committee waived the requirement for patient informed consent.

To ensure the clinical reliability of the ground truth, both datasets were annotated using a multi-expert consensus protocol. Initially, two experienced ultrasound physicians independently delineated the pixel-level contours. Any significant discrepancies were subsequently resolved by a senior associate chief physician, who made the final adjudication by jointly reviewing the ultrasound features and pathological records. This consensus mechanism guarantees the objectivity of our evaluation benchmark.

### 3.2 ESPD-Net framework architecture

**Figure 1:**
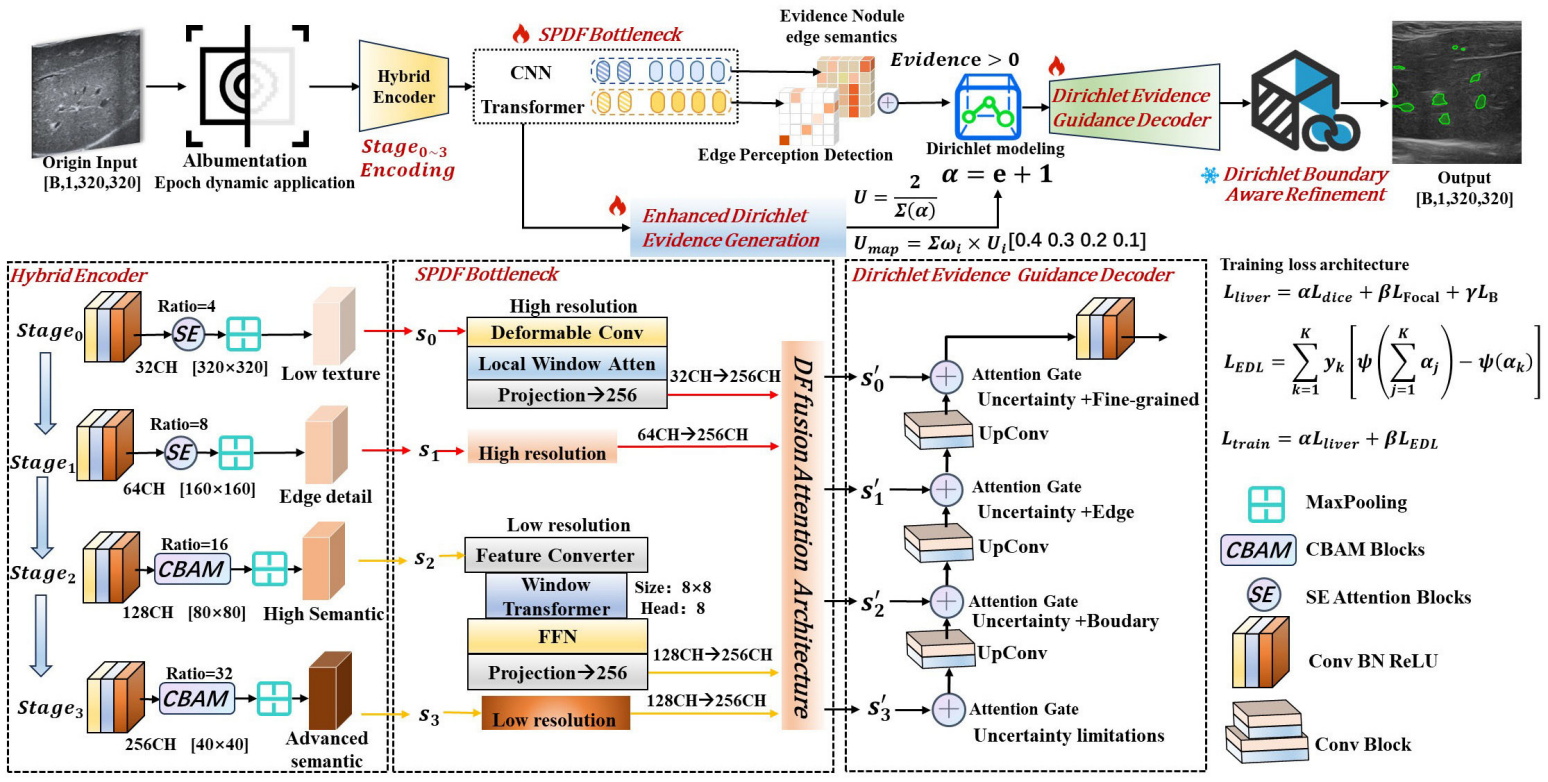
ESPD-Net framework architecture.

To demonstrate the transferability and practicality of the entire detector, this paper selects DSRU-Net for nodule detection as the baseline model and constructs the ESPD-Net architecture by integrating innovative features.Its visualization and overall architecture are shown in Figure 1.

The data augmentation process is implemented using the Albumentations library (Buslaev et al., 2020). It effectively mitigates model overfitting on small medical datasets by performing synchronized geometric transformations and pixel enhancement on paired ultrasound images and segmentation labels.

Subsequently, we implement staged downsampling via a hybrid attention encoder spanning *Stage*_0_ to *Stage*_3_, designed to robustly extract multi-scale features. The encoder comprises four stages: *Stage*_0_ and *Stage*_1_ incorporate lightweight Squeeze-and-Excitation (SE) blocks for channel recalibration, specifically targeting low-level textures and edge features. Meanwhile, *Stage*_2_ and *Stage*_3_ integrate Convolutional Block Attention Modules (CBAM) to model both channel and spatial dependencies. In contrast to architectures employing CBAM across all stages (Roy et al., 2018), this hybrid strategy significantly reduces computational overhead. It strategically enhances semantic feature acquisition while preserving the representational capacity essential for downstream processing.

Following feature extraction, the feature maps are fed in parallel into the corresponding SPDF bottleneck layers, where adaptive fusion is achieved via a probability-density-guided attention mechanism. The fused features are subsequently processed by the DEGD decoder, which employs a multi-scale evidence pyramid to generate Dirichlet concentration parameters and uncertainty maps. During the upsampling phase, this uncertainty information performs evidential modulation on the skip-connection features. This mechanism effectively suppresses unreliable features while reinforcing regions supported by strong evidence, thereby enabling adaptive decoding. Finally, the coarse segmentation output and the uncertainty map are forwarded to the DBAR boundary refinement module. By utilizing the uncertainty map as a spatial weighting factor for iterative refinement, the module successfully achieves the joint modeling of nodule edge semantics and uncertainty probabilistic features. Comprehensive implementation details and the mathematical principles underpinning this architecture are elaborated in Sections 3.3–3.5.

### 3.3 Semantic-probabilistic dual-path fusion (SPDF) bottleneck

**Figure 2:**
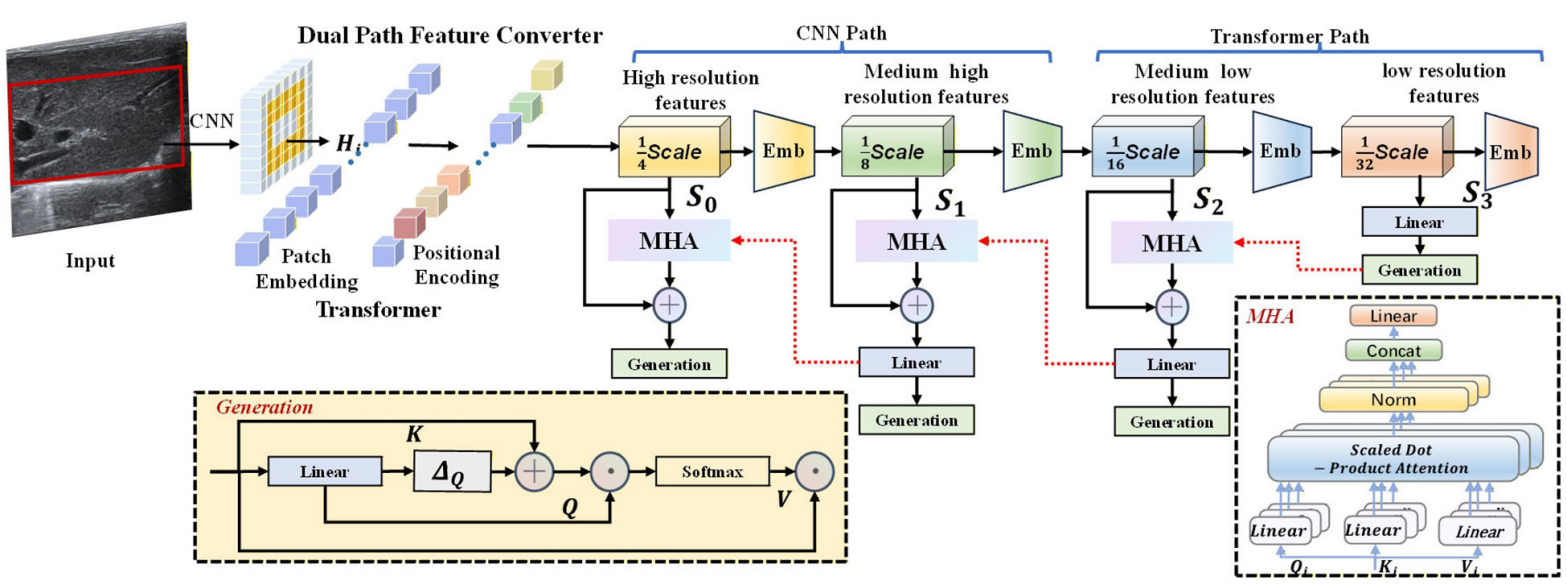
Semantic-probabilistic dual fusion bottleneck.

The complexity of diffuse hepatic fibrosis nodules manifests at two levels: the heterogeneous distribution of fibrous tissue within nodules gives rise to diverse echo intensity variations, while the gradual transition between nodule margins and normal hepatic parenchyma renders boundary localization highly challenging. More critically, the significant grayscale overlap between fibrotic nodules and surrounding normal parenchyma in ultrasound imaging in clinical practice. This necessitates segmentation models capable of both accurately identifying semantic features of lesions and quantifying boundary uncertainty without presupposing clear feature-space separation. Traditional adaptive spatial feature fusion (ASFF) relies on simple weighted summation, which, despite its effectiveness in local feature extraction, lacks the capacity for global uncertainty modeling and is particularly vulnerable to the interference caused by grayscale overlap. Conversely, employing a Transformer architecture throughout incurs an *O*(*N*^2^) computational complexity that becomes prohibitive under high-resolution inputs. To address these limitations, we propose the SPDF module, which achieves deep integration of semantic understanding and probabilistic modeling through a resolution-adaptive parallel dual-path architecture, as illustrated in Figure 2.

#### 3.3.1 Extracting features path

The semantic feature extraction path of SPDF employs deformable convolutions to process high resolution feature maps. It can adaptively adjust the shape of its receptive fields, overcoming the limitations of traditional regular convolution kernels. This enables the precise capture of irregular morphologies in liver fibrosis nodules, such as reticular or cord-like fibrous septa.

The probabilistic path employs a window based self-attention mechanism specifically designed for low resolution feature maps. The rationale for applying Transformers at this stage is twofold: first, the reduced spatial dimensions render the computational cost of self-attention manageable; second, low-resolution features encode richer semantic abstractions that are well suited for global context modeling. By computing self-attention within local windows, this approach effectively captures long-range dependencies whilereducing the computational complexity from *O*(*N*^2^) to *O*(*N* × *W*^2^), where *N* denotes the total number of spatial tokens and *W* represents the window size.

#### 3.3.2 Fusion mechanism

The core innovation of SPDF lies in leveraging the probability density distribution generated by the probabilistic path to guide adaptive enhancement of semantic features. Specifically, the probability density is derived from the variance of the Transformer's output features, reflecting the model's degree of certainty at different spatial locations. Based on this density, a probabilistic attention map is generated to modulate the semantic features:


$$\begin{array}{l} {\text{F}}_{\text{guided}}={\text{F}}_{\text{sem}}⊗\left(1+\alpha \cdot \sigma \left({\text{A}}_{\text{prob}}\right)\right) \end{array}$$ (1)

where A_prob_ denotes the probabilistic attention map, α is a learnable modulation parameter, and σ(·) represents the Sigmoid activation function. As formulated in Equation 1, this mechanism selectively enhances semantic feature responses at nodule centers—where the model exhibits high certainty—while preserving the original representations at ambiguous boundaries, thereby preventing artifacts induced by over-enhancement. From a clinical perspective, this design corresponds to adaptive image quality processing: fully exploiting semantic cues in regions with clear acoustic interfaces while exercising prudence in areas compromised by acoustic shadowing. Finally, integration is achieved through a feedforward network (FFN) with residual connections as formulated in Equation 2:


$$\begin{array}{l} {\text{s}}_{i}^{\text{final}}=\text{FFN}\left(\text{Concat}\left[{\text{s}}_{i}^{\text{sem}},{\text{s}}_{i}^{\text{prob}}\right]\right)+{\text{s}}_{i}^{\text{residual}} \end{array}$$ (2)

**Figure 3:**
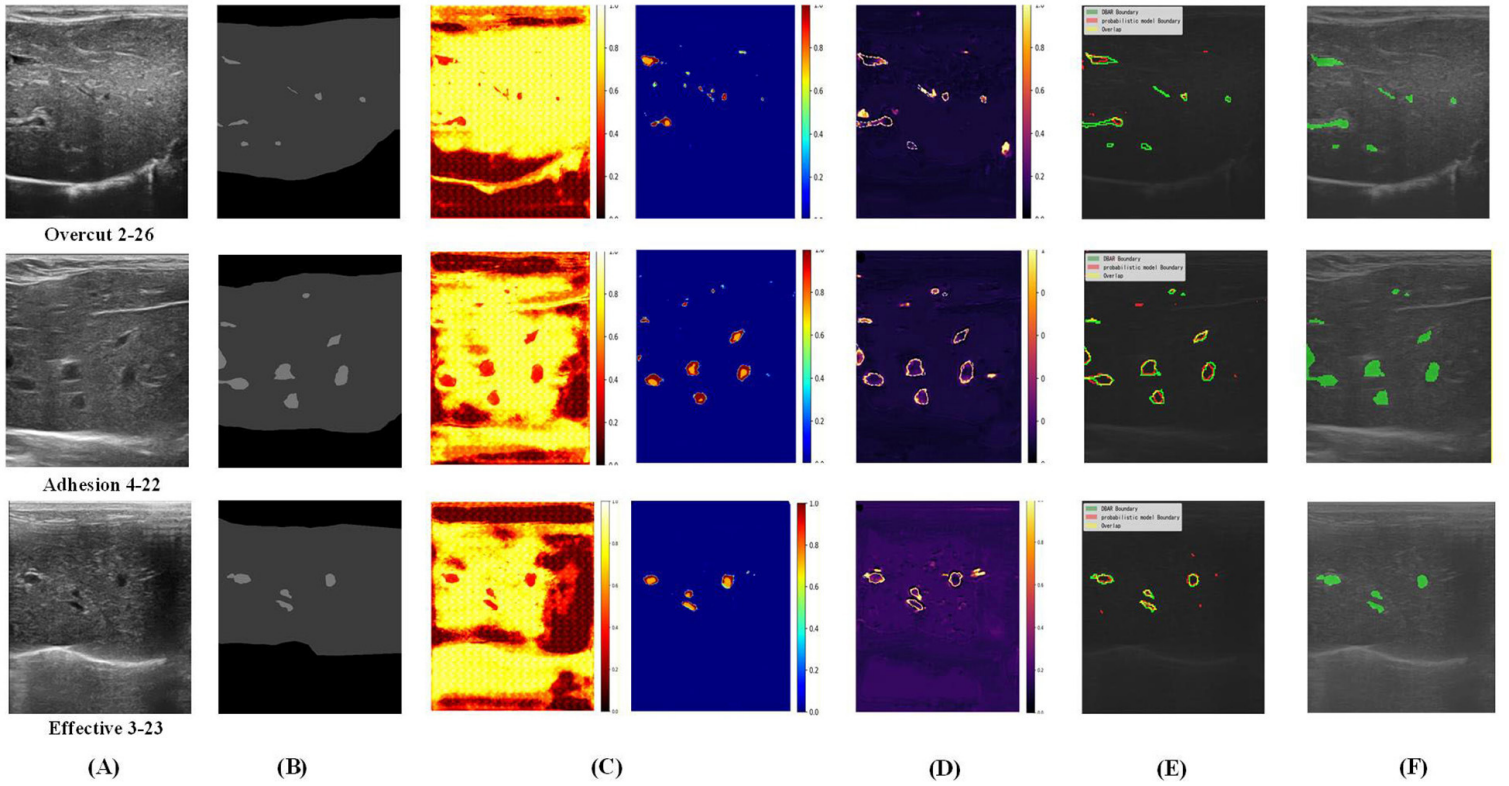
Uncertainty modeling and boundary quality visualization of DEGD and DBAR. **(A)** Origin, **(B)** label, **(C)** uncertainty map *S*_0_→*s*_3_, **(D)** predict entropy, **(E)** DBAR comparison, and **(F)** ESPD-Net (full).

The FFN enables the learning of complex nonlinear interactions between semantic and probabilistic features, while the residual connection ensures the preservation of original information. By dynamically recalibrating feature weights through this probability-guided fusion, SPDF effectively mitigates the interference caused by significant grayscale overlap, reducing the dependency of conventional methods on strict feature separability. This fusion strategy enables SPDF to simultaneously capture local texture patterns and global distribution characteristics of fibrotic lesions, achieving continuous-spectrum modeling from subtle textural alterations to distinct nodule formation. The detailed visualization can be seen in Figure 3.

### 3.4 Dirichlet evidential-guided decoder (DEGD)

The fundamental challenge in segmenting diffuse hepatic fibrosis nodules lies in the inherent uncertainty of their boundaries. Traditional segmentation networks produce deterministic probability estimates via Softmax outputs. Rather than treating uncertainty as a *post-hoc* diagnostic metric, the proposed DEGD decoder actively internalizes evidential theory (He et al., 2025) into the network architecture. It transitions uncertainty from a passive output to an explicit guiding signal for intermediate feature reconstruction.

#### 3.4.1 Second-order probabilistic modeling via the Dirichlet distribution

DEGD employs subjective logic and Dempster-Shafer evidence theory, introducing the Dirichlet distribution as a conjugate prior within a Bayesian framework. For binary classification segmentation, the category probability *p* = [*p*_0_, *p*_1_] for each pixel is no longer a deterministic value but a random variable following the Dirichlet distribution Dir(p|**α**), where the concentration parameter **α** = [α_0_, α_1_] can be interpreted as the cumulative evidence strength. The key uncertainty metric is defined as formulated in Equation 3.


$$\begin{array}{l} U=\frac{K}{S},\;S=\overset{K}{\underset{k=1}{\sum }}\alpha_{k} \end{array}$$ (3)

where *K* = 2 denotes the number of classes and *S* represents the total evidence strength. This formulation offers an intuitive physical interpretation:

- **High-evidence regions** (*S* ≫ *K*): in areas such as the homogeneous hypoechoic zone of the nodule core, multi-source evidence consistently supports foreground classification. Consequently, *U* → 0, indicating high model confidence;
- **Low-evidence regions** (*S* → *K*): in areas such as gradual transition boundaries, the infiltration of fibrous tissue leads to conflicting evidence. Consequently, *U* → 1, indicating high model uncertainty.

The expected estimate of the class probability is given by *p*_*k*_ = α_*k*_/*S*. An advantage of this theoretical framework is the explicit distinction between scenarios yielding identical probabilities: when two pixels both exhibit *p* ≈ 0.5, a high-evidence pixel (large *S*) represents a “confident boundary,” whereas a low-evidence pixel (small *S*) signifies an “ambiguous region.” This distinction is explicitly quantified by *U*, providing the basis for subsequent adaptive processing.

#### 3.4.2 Evidence-guided fusion and attention generation

**Figure 4:**
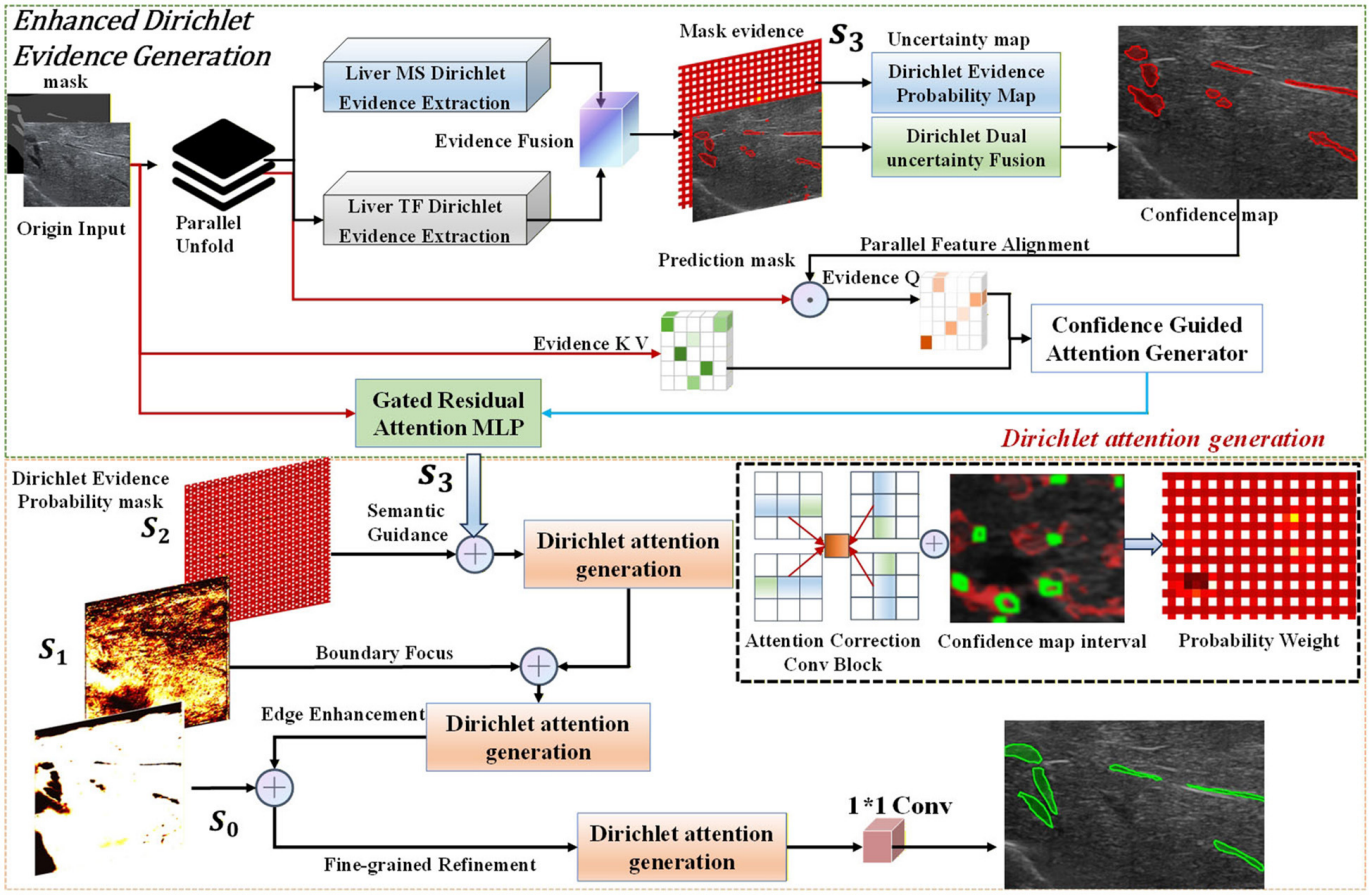
Dirichlet evidential-guided decoder.

As illustrated in Figure 4, the DEGD achieves multi-source extraction, fusion, and adaptive decoding of evidence. This mechanism integrates Dirichlet evidential theory into the attention module, utilizing the quantified evidential confidence to directly guide feature selection. A dual-branch architecture operates in parallel to extract multi-scale spatial evidence and global semantic evidence, subsequently generating mask evidence via adaptive fusion. This process yields three pivotal outputs: the prediction mask provides preliminary segmentation results; the Dirichlet evidential probability map quantifies the uncertainty of the class distribution; and the uncertainty map directly reflects the predictive reliability of each pixel.

Via parallel feature alignment, uncertainty information is transformed into a confidence map, serving as a spatial weighting distribution for subsequent attention guidance. Then, this confidence information is encoded into key-value pairs through the Evidence KV path and fed into the Gated Residual Attention MLP alongside query vectors derived from the prediction mask. As formulated in Equation 4:


$$\begin{array}{l} {\text{A}}_{guided}=\text{MLP}\left({\text{Q}}_{pred}⊕{\text{K}}_{conf}⊕{\text{V}}_{evidence}\right) \end{array}$$ (4)

where Q_*pred*_ encodes regions requiring refinement, K_*conf*_ indicates trustworthy feature locations, and V_*evidence*_ supplies feature importance weights. This design enables the network to selectively focus on strong evidential features within high-confidence regions while simultaneously suppressing noise interference in low-confidence areas.

The bottom path achieves evidence-driven progressive refinement by integrating semantic guidance (S_3_) from the SPDF, the Dirichlet evidential probability mask (S_2_), and edge features derived from the boundary enhancement module as formulated in Equation 5. The Dirichlet Attention Generation module employs a cross-attention mechanism to fuse these complementary information cues:


$$\begin{array}{l} {\text{A}}_{Dir}=\text{CrossAttn}\left({\text{S}}_{2},{\text{S}}_{3},{\text{E}}_{boundary}\right) \end{array}$$ (5)

#### 3.4.3 Evidence-modulated skip connections

In standard U-Net architectures, skip connections directly concatenate encoder features with decoder representations. While preserving spatial details, this operation risks propagating irrelevant background noise and imaging artifacts into the decoding pathway. To mitigate this, we employ the derived Dirichlet evidential attention map, A_*Dir*_, to explicitly modulate the skip connections prior to feature fusion.

Let $F_{input}$ denote the spatial features extracted from the corresponding encoder stage. The modulated feature $F_{corrected}$ is formulated as Equation 6:


$$\begin{array}{l} F_{corrected}=F_{input}⊙\left(1+\alpha \cdot {\text{A}}_{Dir}\right) \end{array}$$ (6)

where ⊙ denotes element-wise multiplication and α is a learnable scaling parameter. This residual-based soft enhancement prevents gradient instability during early training phases while enabling the evidential attention map to dynamically recalibrate the spatial feature responses. Following the modulation, $F_{corrected}$ is processed by a 1 × 1 convolution for channel reduction and then propagated to the subsequent decoding block. Crucially, because A_*Dir*_ is fundamentally driven by the Dirichlet concentration parameters (representing accumulated evidence), this operation mathematically couples the network's epistemic uncertainty with its feature reconstruction process. Through this mechanism, the network translates the quantified evidence strength into spatial modulation weights, explicitly suppressing representations in low-evidence regions while retaining reliable semantic features.

The core contribution of this structural design is the shift of uncertainty from a passive diagnostic indicator to an active control mechanism. This allows the model to dynamically tailor its feature propagation strategy according to its internal epistemic state, achieving genuine adaptive decoding and distinguishing ESPD-Net from conventional *post-hoc* uncertainty methods.

### 3.5 Dirichlet boundary-aware refinement (DBAR)

**Figure 5:**
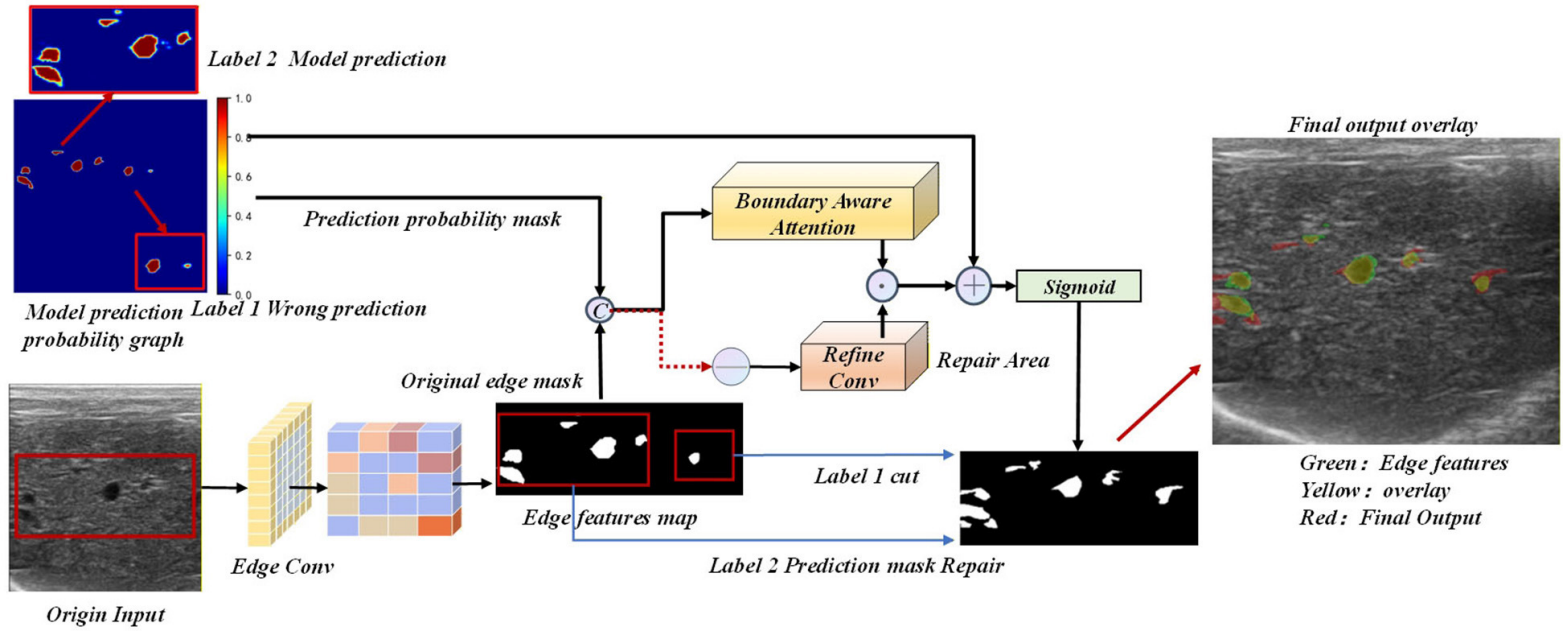
Dirichlet boundary-aware refinement.

In ultrasound imaging of diffuse liver fibrosis, lesion boundaries are inherently ambiguous due to the continuous pathological spectrum extending into the surrounding parenchyma, compounded by acoustic shadowing and attenuation artifacts. Conventional segmentation networks often produce overly smoothed or geometrically inconsistent predictions in these transition zones. To address this limitation, we propose the DBAR module, as illustrated in Figure 5. This module achieves precise geometric boundary correction by explicitly integrating raw image priors, deep semantic features, and the quantified Dirichlet uncertainty.

In the physical path, the DBAR module applies the Sobel operator to the raw ultrasound image to capture abrupt acoustic impedance changes. Concurrently, a learnable edge convolution network is employed on high-level features to extract semantic boundaries. By adaptively fusing these representations, the network outputs a comprehensive edge feature map, E_*boundary*_, ensuring reliable edge detection across varying imaging conditions.

#### 3.5.1 Uncertainty-guided selective refinement

Rather than applying global refinement indiscriminately, DBAR utilizes a selective refinement mechanism strictly governed by the network's epistemic uncertainty. The boundary attention map, A_*boundary*_, is computed by aggregating three distinct inputs:

1. **Prediction uncertainty (U):** the explicit uncertainty map generated by the preceding DEGD module. High values inherently localize regions corrupted by acoustic shadows or motion artifacts.
2. **Probabilistic ambiguity:** derived from the coarse prediction mask M_*coarse*_, targeting pixels with prediction probabilities near 0.5 to locate decision boundaries.
3. **Edge saliency (E_*boundary*_):** the fused dual-path edge features quantifying the structural likelihood of boundaries.

These components are concatenated and processed via cascaded convolutional layers to generate A_*boundary*_. This operation utilizes the Dirichlet uncertainty map directly as a spatial weighting factor. It directs the refinement network to focus its corrective capacity exclusively on highly uncertain regions and structural edges, while preserving the features in high-confidence areas.

The final pixel-level correction is executed via a residual learning formulation as Equation 7.


$$\begin{array}{l} {\text{M}}_{refined}={\text{M}}_{coarse}+{\text{A}}_{boundary}⊙\Delta \text{M} \end{array}$$ (7)

where ΔM denotes the residual correction map predicted by the refinement convolutions, and ⊙ represents element-wise multiplication. In this formulation, the attention map A_*boundary*_ functions as a spatial gate. For high-confidence regions where the attention score approaches zero (A_*boundary*_ → 0), the residual term is gated out, safely preserving the initial prediction (M_*refined*_ ≈ M_*coarse*_). Conversely, in highly uncertain boundary zones, A_*boundary*_ activates the residual correction ΔM. As illustrated in the “Repair Area” of Figure 5, this gated residual mechanism addresses two specific error typologies: (1) False-negative regions (e.g., missed lesion edges with strong physical boundaries but low M_*coarse*_) are rectified via positive residual values generated in ΔM; (2) False-positive regions (e.g., acoustic shadows with high uncertainty but lacking structural edge support) are suppressed via negative residual penalties in ΔM. This mathematical formulation ensures that the final segmentation is rigorously modulated by both structural priors and the model's epistemic uncertainty.

### 3.6 Loss function design

#### 3.6.1 Liver loss

The primary task loss, $L_{liver}$, is tailored to the specific characteristics of liver fibrosis nodule segmentation by integrating three complementary terms. As formulated in Equation 8.


$$\begin{array}{l} L_{liver}=\alpha L_{Dice}+\beta L_{Focal}+\gamma L_{B} \end{array}$$ (8)

where α, β, and γ are empirical balancing weights.

The Dice loss ($L_{Dice}$) ensures global structural consistency and addresses the severe class imbalance inherent to this task, where fibrosis nodules typically occupy merely 3%–8% of the anatomical field. The Focal loss ($L_{Focal}$) down-weights easy-to-classify background pixels, compelling the network to focus on boundary transition zones. Finally, the Boundary loss ($L_{B}$) explicitly optimizes contour accuracy by formulating boundary segmentation as a distance regression task, thereby imposing strict spatial constraints on morphological fidelity.

#### 3.6.2 Dirichlet evidential loss

The evidential loss $L_{EDL}$ is grounded in Subjective Logic and optimizes evidence generation by evaluating the expected cross-entropy risk over the predicted Dirichlet distribution. As formulated in Equation 9.


$$\begin{array}{l} L_{EDL}=\overset{K}{\underset{k=1}{\sum }}y_{k}\left[\psi \left(\overset{K}{\underset{j=1}{\sum }}\alpha_{j}\right)-\psi \left(\alpha_{k}\right)\right] \end{array}$$ (9)

where ψ(·) denotes the digamma function, *y*_*k*_ is the one-hot encoded ground-truth label for class *k*, and α_*k*_ is the predicted Dirichlet concentration parameter.

The gradient mechanism of $L_{EDL}$ acts as an explicit evidence regulator. It encourages evidence accumulation for the correct ground-truth class while implicitly penalizing the generation of misleading evidence for incorrect classes. This structural formulation effectively drives the model to quantify its epistemic state, proactively expressing high uncertainty in ambiguous regions rather than producing overconfident, erroneous predictions.

## 4 Results

### 4.1 Model and experiment implementation details

In this study, the proposed ESPD-Net and all baseline models were implemented in PyTorch and trained on a single NVIDIA GeForce RTX 4090 GPU, with AMP enabled to accelerate training. During preprocessing, ultrasound images were resized to 320 × 320 pixels as single-channel grayscale inputs using the Albumentations library.

For optimization, this paper utilized the AdamW optiizer with an initial learning rate of 1 × 10^−4^, a weight decay of 1 × 10^−5^, and a batch size of 4. The maximum number of training epochs was set to 256. To dynamically adjust the learning rate, a ReduceLROnPlateau scheduler (patience = 5, decay factor = 0.5) was employed, alongside an early stopping mechanism (patience = 20) to prevent overfitting. Finally, the composite loss function defined in the methodology section was uniformly applied across all model training.

To ensure the reliability of experimental results and eliminate incidental variance, all comparative and ablation experiments were independently repeated five times using different random seeds. To verify that the performance improvements achieved by ESPD-Net are statistically significant, we performed a paired permutation test with 1,000 resamplings on the test set to compare our model against the best-performing baseline (BACA-Net). This non-parametric method is independent of data distribution assumptions, yielding a highly rigorous evaluation. In all quantitative result tables, an asterisk (*) explicitly denotes performance gains that are statistically significant (*p* < 0.05).

### 4.2 Quantitative evaluation

#### 4.2.1 Comparison with state-of-the-art methods

Table 1 summarizes the intra-domain quantitative evaluation of all models on both the mouse and clinical datasets. Due to the heightened complexity, acoustic artifacts, and pathological heterogeneity inherent to real-world clinical ultrasound, a general performance degradation is observed across all comparative methods when evaluated on the clinical dataset. Nevertheless, ESPD-Net consistently yields the highest performance across all metrics on both benchmarks. On the more challenging clinical dataset, our method maintains a significant margin of improvement (*p* < 0.05) over state-of-the-art approaches.

**Table 1:** Quantitative comparison of the proposed ESPD-Net and baseline models on the mouse and clinical datasets.

Model	Dataset	Loss	Precision	Sensitivity	IOU	DICE	Val-dice	Boundary-F1	HD95	ASSD
		(↓)	(↑)	(↑)	(↑)	(↑)	(↑)	(↑)	(↓)	(↓)
DSRU-Net (baseline)	Mouse	0.282 ± 0.015	0.704 ± 0.018	0.627 ± 0.016	0.455 ± 0.015	0.612 ± 0.017	0.623 ± 0.019	0.589 ± 0.019	16.04 ± 2.15	5.47 ± 0.25
	clinic	0.316 ± 0.018	0.685 ± 0.024	0.595 ± 0.019	0.438 ± 0.018	0.574 ± 0.020	0.587 ± 0.023	0.542 ± 0.022	16.92 ± 2.35	5.72 ± 0.32
Dense-PSP U-Net	Mouse	0.320 ± 0.012	0.785 ± 0.014	0.715 ± 0.015	0.612 ± 0.012	0.723 ± 0.013	0.705 ± 0.014	0.672 ± 0.017	10.11 ± 1.65	4.15 ± 0.35
	Clinic	0.349 ± 0.015	0.735 ± 0.016	0.685 ± 0.018	0.599 ± 0.015	0.697 ± 0.016	0.715 ± 0.017	0.625 ± 0.021	11.43 ± 2.32	4.48 ± 0.42
Multi-decoder U-Net	Mouse	0.260 ± 0.010	0.790 ± 0.011	0.774 ± 0.011	0.660 ± 0.010	0.750 ± 0.011	0.732 ± 0.012	0.728 ± 0.013	8.74 ± 0.80	3.78 ± 0.15
	Clinic	0.280 ± 0.012	0.727 ± 0.013	0.753 ± 0.013	0.612 ± 0.012	0.709 ± 0.013	0.682 ± 0.014	0.684 ± 0.015	9.46 ± 0.94	3.95 ± 0.20
BACA-Net	Mouse	0.207 ± 0.008	0.820 ± 0.008	0.813 ± 0.011	0.690 ± 0.008	0.812 ± 0.009	0.834 ± 0.009	0.772 ± 0.010	5.90 ± 0.45	3.27 ± 0.08
	Clinic	0.246 ± 0.010	0.765 ± 0.010	0.745 ± 0.014	0.649 ± 0.010	0.747 ± 0.012	0.765 ± 0.012	0.735 ± 0.013	7.42 ± 0.51	3.74 ± 0.12
**ESPD-Net (ours)**	Mouse	**0.190** ± 0.006	**0.912** ± 0.005	**0.835** ± 0.006	**0.747** ± 0.007	0.855^*^± 0.005	**0.874** ± 0.006	0.812^*^± 0.009	3.25^*^± 0.28	2.17^*^± 0.04
	Clinic	**0.225** ± 0.008	**0.873** ± 0.007	**0.822** ± 0.009	**0.734** ± 0.009	0.846^*^± 0.011	**0.866** ± 0.010	0.798^*^± 0.012	3.86^*^± 0.51	2.61^*^± 0.06

(*) denotes statistically significant improvements (*p* < 0.05, evaluated via a paired permutation test with 1,000 resamples against the BACA-Net). Bold values indicate the best performance.

#### 4.2.2 Feature representation and region accuracy

Table 1 demonstrates ESPD-Net's quantitative superiority across region-level metrics, achieving the highest IoU (0.747) and DICE (0.855) on the mouse dataset. This substantial gain over spatial pyramid baselines (IoU: 0.612) is primarily attributed to the proposed SPDF module. Unlike traditional pyramid pooling that concatenates multi-scale features, SPDF dynamically modulates feature weights via spatial probability density, effectively preventing noise integration within heterogeneous diffuse nodule textures. Furthermore, ESPD-Net achieves a remarkable Precision of 0.912, significantly outperforming Multi-Decoder U-Net (0.790). This stark reduction in false-positives is driven by the DEGD module, which leverages Dirichlet evidential uncertainty to systematically suppress unreliable feature propagation.

#### 4.2.3 Boundary delineation and error reduction

The DBAR module's efficacy is most evident in spatial distance metrics. Because HD95 is inherently sensitive to distant spatial outliers, its substantial reduction (from 5.90 to 3.25 on mouse data, and 7.42 to 3.86 on clinical data) quantitatively proves DBAR's capability to prune severe, localized false-positives. While BACA-Net relies on fixed edge patterns (resulting in HD95 of 5.90), ESPD-Net utilizes the quantified Dirichlet uncertainty as an explicit spatial prior to dynamically gate refinement weights. This mechanism effectively suppresses distant acoustic artifacts while preserving true lesion edges, thereby achieving peak Boundary-F1 scores (0.812 and 0.798) and rigorous geometric alignment.

#### 4.2.4 Clinical robustness and architectural stability

Evaluating the model independently on both mouse and clinical datasets rigorously tests its architectural stability. While conventional baselines suffer noticeable performance degradation when applied to the challenging clinical benchmark, ESPD-Net maintans a high DICE score of 0.846. This consistent multi-dataset performance mathematically validates the effect of the DEGD. By preventing the network from overconfidently overfitting to dataset-specific imaging artifacts, ESPD-Net achieves clinical applicability and stable feature extraction capabilities across diverse ultrasound distributions.

### 4.3 Qualitative visual analysis

**Figure 6:**
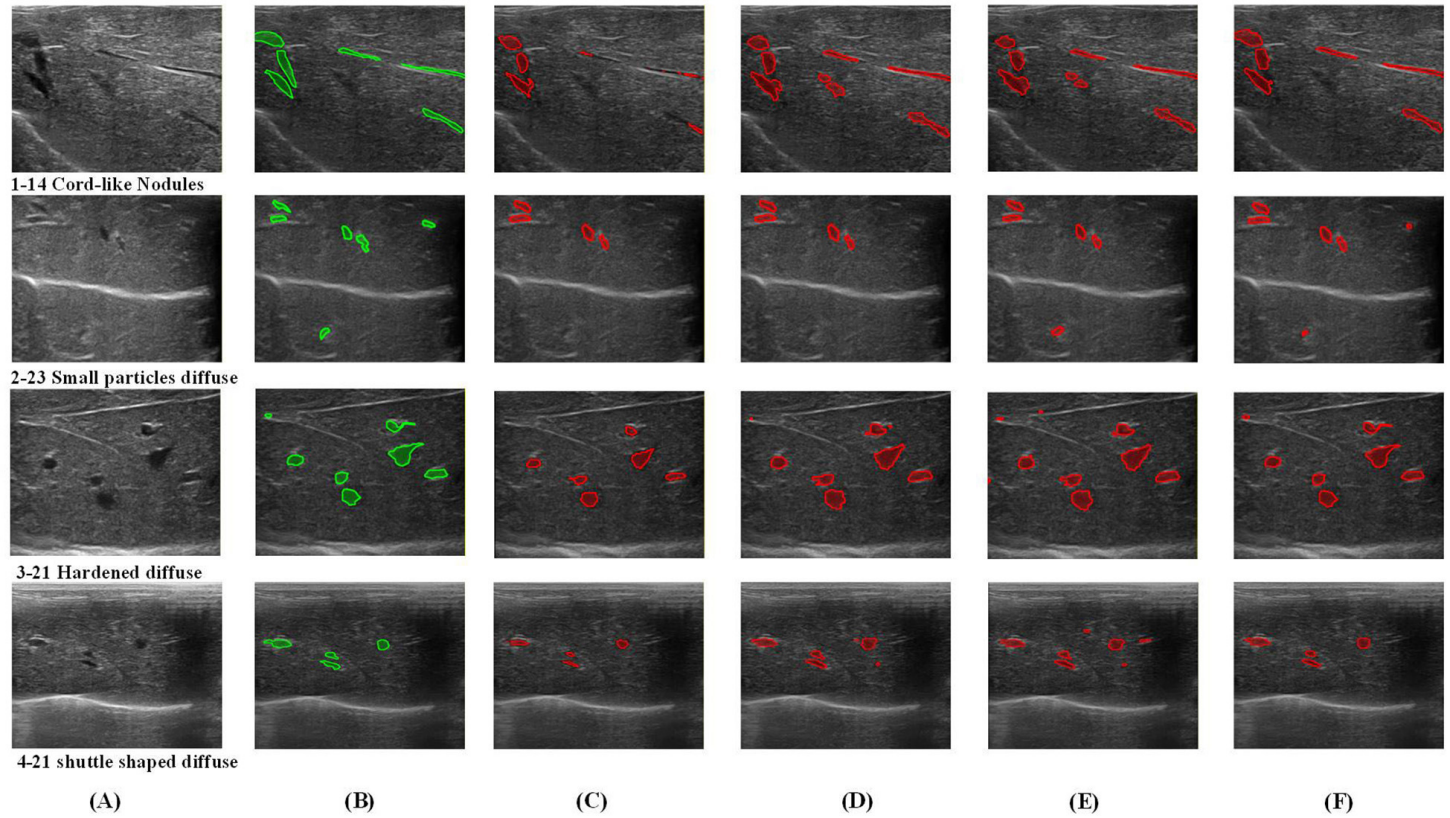
Comparison of effects of different models. **(A)** Origin, **(B)** mask, **(C)** Dense-PSP-UNet, **(D)** multi-decoder U-Net, **(E)** BACA-Net, and **(F)** ESPD-Net (ours).

Figure 6 presents a comparative visualization of segmentation results across four representative scenarios, where green denotes the ground truth (Mask) and red denotes model predictions. These scenarios test the models' capabilities in preserving slender morphology, small target recall, topological separation, and boundary precision under varying contrast conditions.

#### 4.3.1 Cord-like nodules

As shown in the first row of Figure 6, the images display cord-like liver fibrosis nodules In this scenario, baseline methods suffer significantly from missed detections of small targets and spurious false-positives. Dense-PSP U-Net (Column C) fails to capture several fragments and exhibits poor localization. BACA-Net (Column E) misses the far-right fragment slender fragments and introduces distinct false-positive noise artifacts on the background. In contrast, ESPD-Net achieves a superior morphological match with the Mask, preserving the critical features of these slender, fragmented targets. This is attributed to the SPDF frontend: even if nodule density is globally low, the high contrast against the background triggers adaptive attention enhancement, preserving critical details that are lost during standard downsampling.

#### 4.3.2 Small particles diffuse nodules

As presented in the second row of Figure 6, widely dispersed, low-contrast, small-particle nodules rigorously test the global perception capabilities of the models. Multi-Decoder U-Net detects some targets but is heavily corrupted by jagged false-positive noise. BACA Net detected missing targets, but one nodule was not detected due to its small size and distance from the lesion concentration area. ESPD Net captures more comprehensive fine-grained nodules. This success demonstrates the efficacy of the SPDF's Transformer path in capturing global features, enabling detected nodules to structurally amplify the feature responses of other distant potential target regions. This visual recall is the physical basis for ESPD-Net's remarkable Sensitivity (0.835) and Precision (0.912).

#### 4.3.3 Massive dense distribution modules

As shown in the third row of Figure 6, the images exhibit massive dense distribution nodules. It challenges the capability for topological preservation. With minimal inter-nodular spacing, DSRU-Net and Dense-PSP exhibit severe adhesion artifacts. Multi-Decoder partially mitigates this via ensemble averaging but retains significant erroneous fusions, while BACA-Net offers only limited improvement through channel attention. ESPD-Net maintains strict topological consistency, accurately separating independent nodules even when they are spatially adjacent. This success is a direct consequence of the DEGD module: by leveraging quantified Dirichlet uncertainty to suppress feature propagation within the narrow, ambiguous transition gaps between nodules, the network successful forces topological separation. This precise boundary definition is a key reason for the exceptional reduction in ASSD and HD95 (from 5.90 to 3.25) observed in Table 1.

#### 4.3.4 Fusiform low-contrast nodules

The fourth row of Figure 6 illustrates fusiform nodules that exhibit low contrast against the background. It tests the limits of boundary precision. Along gradient boundaries characterized by extremely low contrast, baseline methods exhibit significant deviations of 8–12 pixels. Multi-Decoder's variance estimation yields jagged boundary artifacts, while BACA-Net's boundary detection detected multiple non nodule noises. ESPD-Net restricts boundary error to 4 pixels via the sample-adaptive mechanism of DBAR. By optimizing based on the evidence distribution of the specific instance rather than memorized training patterns, the model demonstrates inherent robustness to unseen boundary morphologies.

### 4.4 Ablation experiment

#### 4.4.1 Ablation study settings and module dependencies

All ablation experiments in this section are conducted on the mouse dataset due to its larger sample size, ensuring robust statistical validation. The ablation study systematically isolates and evaluates the contributions and synergistic effects of each module, demonstrating that ESPD-Net is not merely a concatenation of independent components, but a coupled architecture driven by intrinsic logical dependencies.

Architecturally, the SPDF module extracts the foundational representations prerequisite for DEGD's evidential reasoning, rendering the isolated evaluation of DEGD structurally invalid. The DBAR module functions as an independent, downstream boundary refiner. Driven by these dependencies, this paper employs a progressive ablation strategy across six variants. To rigorously assess the uncertainty-guided mechanisms, we expand the standard evaluation framework to include ECE and UEC. These specific metrics are introduced to quantitatively validate the synergistic impact of our modules on calibration reliability and boundary preservation.

- **Baseline:** serving as the fundamental performance evaluation benchmark, this baseline consists of a stable traditional encoder-decoder and Softmax probabilities. It presents the inherent limitations of standard networks in handling boundary ambiguity and overconfident misclassifications.
- **Baseline + SPDF (establishing the feature foundation):** designed to validate the effectiveness of the SPDF module in feature extraction. By replacing the standard bottleneck layer, By replacing the standard bottleneck layer, it enriches the global and local representations, providing the hierarchical feature prerequisite for subsequent modules.
- **Baseline + DBAR (boundary mechanism):** investigates the independent corrective capability of the boundary module. Stripped of dual-path enhancements and evidential modeling, this configuration directly utilizes DBAR to perform spatial geometric corrections on the raw, flawed baseline predictions, highlighting its contribution to boundary metrics.
- **Baseline + SPDF + DEGD (introducing evidential uncertainty):** demonstrates the regional gains achieved by the Dirichlet theory-based probability-guided decoder. Built upon the dual path feature foundation of SPDF, this variant adopts evidence based confidence learning. It utilizes the uncertainty distribution to explicitly guide the model to adaptively focus on high frequency informative regions, thereby calibrating the Expected Calibration Error (ECE).
- **Baseline + SPDF + DBAR (boundary refinement under enhanced features):** validates the boundary correction capability within an enhanced feature environment. Guided by the richer dual-path features from SPDF, the initial probability distribution output by the bottleneck layer directly drives the DBAR module to perform secondary geometric alignment and detail refinement on the edges of complex lesions.
- **ESPD-Net (the complete framework):** the fully realized network architecture (Baseline + SPDF + DEGD + DBAR). SPDF establishes a substantial feature repository, DEGD outputs high-confidence region-level probability guidance, and DBAR receives these dually optimized features to execute the final boundary refinement. The three modules achieve performance optimization across both regional and boundary metrics.

#### 4.4.2 Analysis of ablation results

The quantitative results of the step wise ablation study on the mouse dataset are detailed in Table 2. Rather than merely listing performance gains, this analysis focuses on aligning the empirical variations in specific metrics with their underlying architectural mechanisms.

- **Impact of SPDF on feature representation:** in the standard Baseline, successive downsampling inevitably causes the loss of critical high-frequency details, yielding a Precision of 0.704. The integration of the SPDF module specifically addresses this by establishing a probability-density-guided feature interaction. This robust global modeling effectively suppresses false-positive predictions caused by feature ambiguity, which is directly reflected in the increase in Precision to 0.812 and IOU to 0.564. This validates that SPDF successfully constructs a reliable feature repository for subsequent decoding.
- **Isolated effectiveness of DBAR on boundary constraints:** observing the Baseline + DBAR configuration provides crucial insight into the module's specific physical role. While this variant achieves only an improvement in regional metrics, it triggers a reduction in spatial distance errors: HD95 drops from 16.04 to 11.85, and ASSD decreases from 5.47 to 4.12. This geometric improvement confirms that DBAR is logically orthogonal to voxel-wise classification; it does not blindly inflate the predicted area but actively corrects structural disconnections and prunes distant spatial outliers.
- **DEGD and evidential stability:** the transition from deterministic predictions to evidential uncertainty modeling is validated by comparing the + SPDF + DBAR and + SPDF + DEGD variants. The DEGD module leverages Dirichlet parameterization to explicitly filter unreliable signals in low-evidence regions. Consequently, the DICE score reaches to 0.841, and Sensitivity reaches 0.807. More importantly, DEGD triggers a systemic contraction in the standard deviations across multiple metrics, such as the SD of DICE drops to 0.008, and the SD of Loss drops to 0.008. This variance reduction objectively proves that evidential reasoning effectively decouples incidental noise, granting the model exceptional convergence stability against heterogeneous clinical features.
- **The ESPD-Net (full):** the quantitative results of the complete ESPD-Net illustrate the sequential architectural dependency among the three proposed modules. As the foundational step, the SPDF frontend extracts robust dense representations, which are prerequisite for the subsequent probabilistic modeling. Utilizing these features, DEGD quantifies the epistemic uncertainty to localize ambiguous tissues, while DBAR subsequently integrates this specific map as a spatial gate for targeted contour refinement. The final complete model framework achieved the optimal performance in the ablation experiment, with DICE of 0.855, accuracy of 0.912, and HD95 reduced to 3.25.

**Table 2:** Ablation study of different components in the proposed ESPD-Net on the mouse dataset.

Ablation configuration	Loss	Precision	Sensitivity	IOU	DICE	Val-dice	Boundary-F1	HD95	ASSD
	(↓)	(↑)	(↑)	(↑)	(↑)	(↑)	(↑)	(↓)	(↓)
Baseline	0.282 ± 0.015	0.704 ± 0.018	0.627 ± 0.016	0.455 ± 0.015	0.612 ± 0.017	0.623 ± 0.019	0.589 ± 0.019	16.04 ± 2.15	5.47 ± 0.25
+ SPDF	0.299 ± 0.016	0.812 ± 0.015	0.700 ± 0.018	0.564 ± 0.014	0.713 ± 0.015	0.695 ± 0.016	0.611 ± 0.017	12.59 ± 1.85	4.65 ± 0.22
+ DBAR	0.312 ± 0.018	0.725 ± 0.017	0.615 ± 0.015	0.475 ± 0.016	0.634 ± 0.014	0.637 ± 0.015	0.627 ± 0.015	11.85 ± 1.15	4.12 ± 0.18
+ SPDF + DBAR	0.283 ± 0.012	0.765 ± 0.013	0.735 ± 0.014	0.601 ± 0.011	0.741 ± 0.012	0.744 ± 0.014	0.695 ± 0.013	8.15 ± 0.85	3.45 ± 0.12
+ SPDF + DEGD	0.219 ± 0.008	0.891 ± 0.008	0.807 ± 0.009	0.728 ± 0.007	0.841 ± 0.008	0.834 ± 0.010	0.757 ± 0.011	5.94 ± 0.65	3.23 ± 0.10
**ESPD-Net (full)**	**0.190** ± **0.004**	**0.912** ± **0.005**	**0.835** ± **0.006**	**0.747** ± **0.005**	**0.855** ± **0.005**	**0.874** ± **0.006**	**0.812** ± **0.006**	**3.25** ± **0.28**	**2.17** ± **0.04**

Results are reported as mean ± standard deviation across five independent runs. Bold values indicate the best performance.

#### 4.4.3 Quantitative validation of uncertainty calibration and boundary refinement

To deeply analyze the reliability of the model's probabilistic outputs and their impact on boundary refinement, we evaluate the Expected Calibration Error (ECE) and Uncertainty-Error Correlation (UEC), with quantitative results presented in Table 3 and corresponding visual mechanisms in Figure 3 (Failed cases: Row 1,2, effective case: Row 3).

**Table 3:** Impact of evidential uncertainty modeling and boundary refinement on network calibration and segmentation accuracy.

Model configuration	HD95	Bound-F1	ASSD	ECE	UEC
	(↓)	(↑)	(↓)	(↓)	(↑)
Baseline + SPDF (Softmax)	12.59	0.611	4.65	11.40%	0.439
+ DEGD (Dirichlet)	5.94	0.757	3.23	5.12%	0.652
**ESPD-Net (ours)**	**3.25**	**0.812**	**2.17**	**3.85%**	**0.727**

Bold values indicate the best performance.

**Analysis of calibration and boundary metrics:** as demonstrated in Table 3, the standard Softmax-based baseline yields a high ECE of 11.40% and a low UEC of 0.439. In conventional deep learning architectures, the Softmax function converts raw network logits into a normalized probability distribution. However, due to its exponential nature, Softmax tends to disproportionately amplify the maximum logit. This inherent characteristic frequently forces the network to generate highly confident probability scores even for ambiguous boundary pixels. Consequently, this overconfidence limits the model's ability to reliably reflect its true predictive uncertainty, directly leading to the observed high calibration error. The integration of the DEGD module mitigates this issue by parameterizing the predictive distribution based on accumulated evidence strength ($S=\underset{K}{\sum }\alpha_{k}$). In pathological transition zones lacking clear structural features, the network collects minimal evidence (*S* ≈ *K*), which translates into higher epistemic uncertainty rather than a forced deterministic prediction. This evidence-based modeling aligns the output confidence more closely with the actual segmentation accuracy, which is quantitatively reflected in the reduction of ECE to 5.12% and the increase of UEC to 0.652.

Furthermore, the DBAR module utilizes this calibrated uncertainty map as a spatial prior to guide contour refinement. By specifically adjusting predictions in high-uncertainty boundary zones, DBAR not only improves spatial geometric metrics (reducing HD95 to 3.25 and ASSD to 2.17) but also contributes to the final reliability of the output probabilities. Consequently, the fully equipped ESPD-Net achieves an ECE of 3.85% and a UEC of 0.727. This indicates that explicitly incorporating epistemic uncertainty into localized geometric correction positively impacts the overall calibration of the network.

**Visualizing the evidence-to-entropy and mechanistic analysis:** Figure 3 visualizes the calibration mechanism across three scenarios. Column (C) maps the dynamic accumulation of Dirichlet evidence (*S*_0_ → *S*_3_), capturing the model's progressive localization of fibrosis targets. From this evidence, Column (D) derives the predictive entropy. Rather than producing diffuse and uninformative gradients, the entropy maps exhibit highly localized, sharp peaks explicitly at ambiguous lesion boundaries where tissue contrast is exceptionally low. This confirms that the DEGD module effectively isolates error-prone regions, laying the foundational spatial prior for subsequent refinement. Column (E) explicitly demonstrates DBAR's targeted intervention by overlaying the initial probabilistic prediction (Red), the final DBAR-refined prediction (Green), and their consensus (Yellow). By correlating the predictive entropy with these geometric corrections, the specific behaviors and limits of the architecture become evident:

- *Effective geometric pruning (row 3, effective 3–23):* the predictive entropy effectively isolates scattered false-positive noise. Utilizing these high-uncertainty signals, DBAR executes targeted pruning–observed as the elimination of isolated red fragments–bringing the refined contours into closer alignment with the anatomical ground truth.
- *Geometric adjustments in high ambiguity (rows 1 & 2):* the “Overcut" and “Adhesion" cases represent challenging scenarios where interstitial tissue mimics nodular characteristics. In these transition gaps, the predictive entropy (D) exhibits high activation due to insufficient structural evidence. While DBAR successfully filters distant background noise, the local ambiguity causes geometric over-adjustment, resulting in the unintended merging of adjacent micro-nodules (Row 1) or incomplete topological separation (Row 2).

**Validation of the uncertainty-error correlation (UEC):** these failure cases inherently validate the high UEC metric (0.727) reported in Table 3. Specifically, the spatial overlap between the high predictive entropy regions in Column (D) and the actual geometric errors in Column (E) demonstrates that the network effectively highlights ambiguous predictions. This consistency confirms that the Dirichlet-quantified uncertainty serves as a reliable indicator for localizing potential segmentation failures.

## 5 Conclusions

This paper presents ESPD-Net, an uncertainty-aware architecture specifically designed for the robust segmentation of diffuse liver fibrosis in ultrasound imaging. By integrating Dirichlet evidential theory into the feature reconstruction process, the network systematically quantifies and leverages epistemic uncertainty to address the inherent ambiguities caused by acoustic artifacts and pathological heterogeneity. the SPDF frontend for multi features extraction, the DEGD module for uncertainty-driven decoding, and the DBAR module for selective boundary refinement.

Extensive evaluations on independent mouse and clinical datasets confirm the architectural stability of ESPD-Net. By penalizing overconfident predictions in unfamiliar feature spaces, the Dirichlet prior ensures consistent performance without overfitting to dataset-specific noise patterns. Future work will focus on validating this evidential framework across larger, multi-center clinical cohorts and extending the uncertainty modeling to 3D volumetric ultrasound analysis.

## Data Availability

The raw data supporting the conclusions of this article will be made available by the authors, without undue reservation.

## References

[B1] Akram M. Adnan M. Ali S. F. Ahmad J. Yousef A. Alshalali T. A. N. . (2025). Uncertainty-aware diabetic retinopathy detection using deep learning enhanced by Bayesian approaches. Sci. Rep. 15:1342. doi: 10.1038/s41598-024-84478-x 39779778 PMC11711487

[B2] Ansari M. Y. Yang Y. Meher P. K. Dakua S. P. (2023). Dense-PSP-UNET: a neural network for fast inference liver ultrasound segmentation. Comput. Biol. Med. 153:106478. doi: 10.1016/j.compbiomed.2022.106478 36603437

[B3] Buslaev A. Iglovikov V. I. Khvedchenya E. Parinov A. Druzhinin M. Kalinin A. A. . (2020). Albumentations: fast and flexible image augmentations. Information 11:125. doi: 10.3390/info11020125

[B4] Chen J. Lu Y. Yu Q. Luo X. Adeli E. Wang Y. . (2021). Transunet: transformers make strong encoders for medical image segmentation. arXiv [Preprint]. arXiv:2102.04306.

[B5] Chen X. Pan L. (2018). A survey of graph cuts/graph search based medical image segmentation. IEEE Rev. Biomed. Eng. 11, 112–124. doi: 10.1109/RBME.2018.2798701 29994356

[B6] Choi H. H. Rodgers S. K. Fetzer D. T. Wasnik A. P. Millet J. D. Morgan T. A. . (2021). Ultrasound liver imaging reporting and data system (US LI-RADS): an overview with technical and practical applications. Acad. Radiol. 28, 1464–1476. doi: 10.1016/j.acra.2020.06.004 32718745

[B7] He Y. Li L. Zhan T. Pun C.-M. Jiao W. Jin Z. . (2025). “Evidential prototype learning for semi-supervised medical image segmentation,” in Proceedings of the 31st ACM SIGKDD Conference on Knowledge Discovery and Data Mining V. 2 (New York, NY: ACM), 908–919. doi: 10.1145/3711896.3736944

[B8] Huang Z. Yang Y. Zhao T. Yang X. (2024). “A noise robust framework via uncertainty guidance for medical image segmentation with noisy label”, in *2024 IEEE International Conference on Multimedia and Expo (ICME)* (IEEE), 1–6. doi: 10.1109/ICME57554.2024.10687399

[B9] Kisseleva T. Brenner D. (2021). Molecular and cellular mechanisms of liver fibrosis and its regression. Nat. Rev Gastroenterol. Hepatol. 18, 151–166. doi: 10.1038/s41575-020-00372-7 33128017

[B10] Liu S. Wang Y. Yang X. Lei B. Liu L. Li S. X. . (2019). Deep learning in medical ultrasound analysis: a review. Engineering 5, 261–275. doi: 10.1016/j.eng.2018.11.020

[B11] Liu X. Zhan Z. Yan M. Zhao J. Song J. Chen Y. Q. . (2017). “Computer-aided cirrhosis diagnosis via automatic liver capsule extraction and combined geometry-texture features”, in 2017 IEEE International Conference on Multimedia and Expo (ICME) (Hong Kong: IEEE), 1–6. doi: 10.1109/ICME.2017.8019551

[B12] Liu Y. Liu X. Wang S. Song J. Zhang J. (2020). A novel method for accurate extraction of liver capsule and auxiliary diagnosis of liver cirrhosis based on high-frequency ultrasound images. Comput. Biol. Med. 125:104002. doi: 10.1016/j.compbiomed.2020.104002 32979541

[B13] Long J. Shelhamer E. Darrell T. (2015). “Fully convolutional networks for semantic segmentation”, in Proceedings of the IEEE Conference on Computer Vision and Pattern Recognition, 3431–3440. doi: 10.1109/CVPR.2015.7298965 27244717

[B14] Lyshchik A. Wessner C. E. Bradigan K. Eisenbrey J. R. Forsberg F. Yi M. . (2024). Contrast-enhanced ultrasound liver imaging reporting and data system: clinical validation in a prospective multinational study in North America and Europe. Hepatology 79, 380–391. doi: 10.1097/HEP.0000000000000558 37548928 PMC11810132

[B15] Ma H.-Y. Lin Y.-H. Wang C.-Y. Chen C.-N. Ho M.-C. Tsui P.-H. . (2016). Ultrasound window-modulated compounding Nakagami imaging: resolution improvement and computational acceleration for liver characterization. Ultrasonics 70, 18–28. doi: 10.1016/j.ultras.2016.04.011 27125557

[B16] Mishra D. Chaudhury S. Sarkar M. Soin A. S. (2018). Ultrasound image segmentation: a deeply supervised network with attention to boundaries. IEEE Trans. Biomed. Eng. 66, 1637–1648. doi: 10.1109/TBME.2018.2877577 30346279

[B17] Oktay O. Schlemper J. Folgoc L. L. Lee M. Heinrich M. Misawa K. . (2018). Attention U-Net: learning where to look for the pancreas. arXiv [Preprint]. arXiv:1804.03999.

[B18] Qin Q. Lin Z. Gao G. Han C. Wang R. Qin Y. . (2025). MBE-UNET: multi-branch boundary enhanced u-net for ultrasound segmentation. IEEE J. Biomed. Health Inform. 30, 575–585. doi: 10.1109/JBHI.2025.3589293 40663661

[B19] Ronneberger O. Fischer P. Brox T. (2015). “U-net: convolutional networks for biomedical image segmentation”, in International Conference on Medical Image Computing and Computer-Assisted Intervention (Cham: Springer), 234–241. doi: 10.1007/978-3-319-24574-4_28

[B20] Roy A. Navab N. Wachinger C. (2018). “Concurrent spatial and channel squeeze & excitation in fully convolutional networks”, in arXiv: Computer Vision and Pattern Recognition (Cham: Springer), doi: 10.1007/978-3-030-00928-1_48

[B21] Senthilkumaran N. Vaithegi S. (2016). Image segmentation by using thresholding techniques for medical images. Comput. Sci. Eng. Int. J. 6, 1–13. doi: 10.5121/cseij.2016.6101

[B22] Shuo H. W. Xiang L. Jingwen Z. Jia L. S. Jian Q. Z. Yan Q. C. . (2016). Learning to Diagnose Cirrhosis via Combined Liver Capsule and Parenchyma Ultrasound Image Features.

[B23] Song Y. Elibol A. Chong N. Y. (2023). “Two-path augmented directional context aware ultrasound image segmentation”, in 2023 IEEE International Conference on Mechatronics and Automation (ICMA) (Harbin: IEEE), 1815–1822. doi: 10.1109/ICMA57826.2023.10215672

[B24] Tan Z. Sun H. Xue T. Gan C. Liu H. Xie Y. . (2021). Liver fibrosis: therapeutic targets and advances in drug therapy. Front. Cell Dev. Biol. 9:730176. doi: 10.3389/fcell.2021.730176 34621747 PMC8490799

[B25] Valanarasu J. M. J. Patel V. M. (2022). “UNEXT: MLP-based rapid medical image segmentation network”, in International Conference on Medical Image Computing and Computer-Assisted Intervention (Cham: Springer), 23–33. doi: 10.1007/978-3-031-16443-9_3

[B26] Wang R. Chen S. Ji C. Fan J. Li Y. (2022). Boundary-aware context neural network for medical image segmentation. Med. Image Anal. 78:102395. doi: 10.1016/j.media.2022.102395 35231851

[B27] Wang S. H. Liu X. Zhao J. Song J. L. Zhang J. Q. Chen Y. Q. (2016). “Learning to diagnose cirrhosis via combined liver capsule and parenchyma ultrasound image features,” in 2016 IEEE International Conference on Bioinformatics and Biomedicine (BIBM) (IEEE), 799–804. doi: 10.1109/BIBM.2016.7822627

[B28] Wu J. Liu F. Sun W. Liu Z. Hou H. Jiang R. . (2024). Boundary-aware convolutional attention network for liver segmentation in ultrasound images. Sci. Rep. 14:21529. doi: 10.1038/s41598-024-70527-y 39278955 PMC11403006

[B29] Wu S. Wei X. Chen X. Ren Y. He J. Pu X. . (2024). “Cross-view mutual learning for semi-supervised medical image segmentation”, in Proceedings of the 32nd ACM International Conference on Multimedia (New York, NY: ACM), 9253–9261. doi: 10.1145/3664647.3680699

[B30] Zhang X. Lau H. C.-H. Yu J. (2025). Pharmacological treatment for metabolic dysfunction-associated steatotic liver disease and related disorders: current and emerging therapeutic options. Pharmacol. Rev. 77:100018. doi: 10.1016/j.pharmr.2024.100018 40148030

[B31] Zheng T. Qin H. Cui Y. Wang R. Zhao W. Zhang S. . (2023). Segmentation of thyroid glands and nodules in ultrasound images using the improved u-net architecture. BMC Med. Imaging 23:56. doi: 10.1186/s12880-023-01011-8 37060061 PMC10105426

[B32] Zhou S. Wang J. Zhang S. Liang Y. Gong Y. (2016). Active contour model based on local and global intensity information for medical image segmentation. Neurocomputing 186, 107–118. doi: 10.1016/j.neucom.2015.12.073

[B33] Zhou X. Chen T. (2024). “BSBP-RWKV: background suppression with boundary preservation for efficient medical image segmentation”, in Proceedings of the 32nd ACM International Conference on Multimedia (New York, NY: ACM), 4938–4946. doi: 10.1145/3664647.3681033

[B34] Zhou X. Wu S. Qiao Y. Guo Y. Qian C. Zhang X. . (2025). Research on multi-objective optimization of medical image segmentation based on frequency domain decoupling and dual attention mechanism. Int. J. Imaging Syst. Technol. 35:e70186. doi: 10.1002/ima.70186

[B35] Zhou Z. Gao A. Zhang Q. Wu W. Wu S. Tsui P.-H. . (2020). Ultrasound backscatter envelope statistics parametric imaging for liver fibrosis characterization: a review. Ultrason. Imaging 42, 92–109. doi: 10.1177/0161734620907886 32100633

[B36] Zhou Z. Siddiquee M. M. R. Tajbakhsh N. Liang J. (2019). Unet++: redesigning skip connections to exploit multiscale features in image segmentation. IEEE Trans. Med. Imaging 39, 1856–1867. doi: 10.1109/TMI.2019.2959609 31841402 PMC7357299

