# Supplementary figures and images for: A novel diffuse liver nodule detector via integrating semantic edge features and probabilistic uncertainty modeling
Source: Front Artif Intell. 2026 Apr 20;9:1801342. doi: 10.3389/frai.2026.1801342 (PMC13136238; doi:10.3389/frai.2026.1801342)

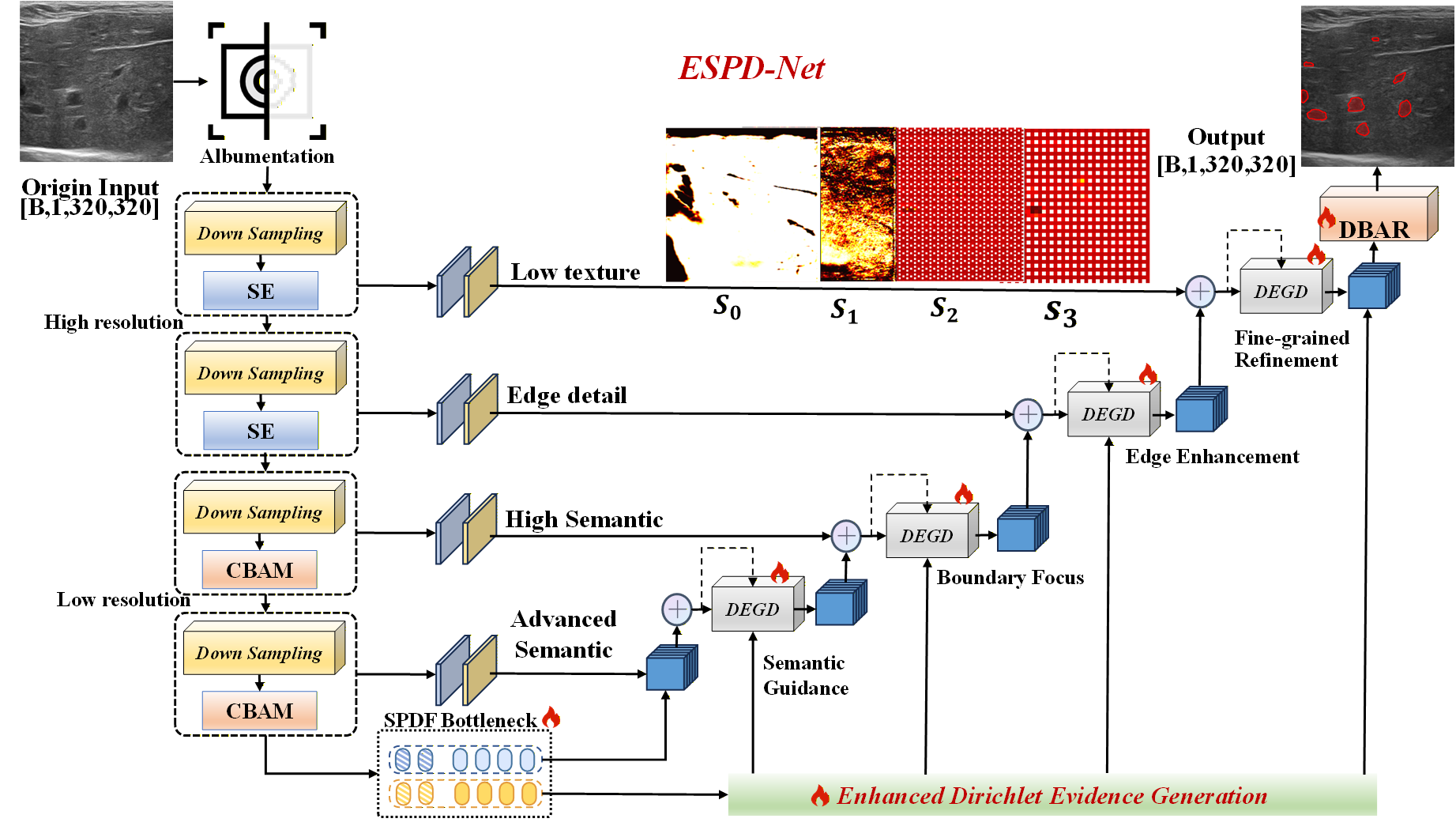

Supplement: Supplementary file 1 [file Image_1.tif]
